# Exploration of the primary antibiofilm substance and mechanism employed by *Lactobacillus salivarius* ATCC 11741 to inhibit biofilm of *Streptococcus mutans*

**DOI:** 10.3389/fcimb.2025.1535539

**Published:** 2025-03-11

**Authors:** Nan Ma, Wei Yang, Bairu Chen, Meihua Bao, Yimin Li, Meng Wang, Xiaopeng Yang, Junyi Liu, Chengyue Wang, Lihong Qiu

**Affiliations:** ^1^ Department of Periodontics, Affiliated Stomatology Hospital of Jinzhou Medical University, Jinzhou, China; ^2^ Collaborative Innovation Center for Health Promotion of Children and Adolescents of Jinzhou Medical University, Jinzhou, China; ^3^ Department of Pedodontics, Affiliated Stomatology Hospital of Jinzhou Medical University, Jinzhou, China; ^4^ Department of Prosthetics, Affiliated Stomatology Hospital of Jinzhou Medical University, Jinzhou, China; ^5^ Jinzhou Medical University, Jinzhou, China; ^6^ Department of Endodontics, School and Hospital of Stomatology, China Medical University, Liaoning Provincial Key Laboratory of Oral Diseases, Shenyang, China

**Keywords:** *Streptococcus mutans*, transcriptomics, metabolomics, biofilm, dental caries, *Lactobacillus salivarius*

## Abstract

**Introduction:**

*Lactobacillus salivarius* serves as a probiotic potentially capable of preventing dental caries both *in vitro* and *in vivo*. This study focused on understanding the key antibiofilm agents and the mechanisms of action of the *Lactobacilli* supernatant against *Streptococcus mutans*.

**Methods:**

*Streptococcus mutans* biofilm was constructed and the cell-free supernatant of *Lactobacillus salivarius* was added. After the biofilm was collected, RNA-seq and qRT-PCR were then performed to get gene information. The influence of temperature, pH and other factors on the supernatant were measured and non-targeted metabolome analysis was performed to analyze the effective components.

**Results:**

The findings indicated that the supernatant derived from *Lactobacillus salivarius* could inhibit the biofilm formation of *Streptococcus* mutans at different times. Through transcriptome analysis, we discovered that the cell-free supernatant reduced biofilm formation, by suppressing phosphoenolpyruvate-dependent phosphotransferase systems along with two ATP-binding cassette transporters, rather than directly affecting the genes that code for glucosyltransferases; additionally, the supernatant was observed to diminish the expression of genes linked to two-component systems, polyketides/non-ribosomal peptides, acid stress response, quorum sensing, and exopolysaccharide formation. Non-targeted LC-MS/MS analysis was employed to discover a variety of potential active compounds present in the cellular filtrate of *Lactobacillus salivarius* that hinder the growth of S. mutans, including phenyllactic acid, sorbitol, and honokiol.

**Discussion:**

In summary, our findings support the evaluation of *Lactobacillus salivarius* as a promising oral probiotic aimed at hindering the formation of biofilms by cariogenic pathogens and the development of dental caries.

## 1 Introduction

Oral health is intricately connected to overall wellbeing. The World Health Organization explicitly identifies oral health as one of the 10 essential components of human health. Dental caries is a common oral condition that poses significant risks to public health. Its condition is a chronic and progressive disease that stems from dental plaques housing cariogenic microorganisms and characterized by demineralization of inorganic substance and decomposition of organics (Lin et al., 2022).

*Streptococcus mutans* is a type of Gram-positive bacterium known for its strong ability to metabolize sucrose, generate acids, and contribute to the formation of cariogenic organisms. It is the main colonizing bacteria in the beginning stage of biofilm formation, which is regarded as the main pathogen of dental caries (Li et al., 2020). Long seen as a common pathogen within the oral microbiome, *S. mutans* was confirmed to be a pivotal microorganism implicated in the progression of dental caries, which utilizes sucrose to produce acid rapidly, creating a local low pH environment to assist the colonization of other cariogenic bacteria, forming a cariogenic biofilm that ultimately generates tooth decay (Wasfi et al., 2012; Liu et al., 2021). Cariogenic biofilm is a highly dynamic and structured microbial community, covered by the extracellular polymeric substance (EPS) matrix on its surface, which comprises various polymers such as extracellular polysaccharides, proteins, and nucleic acids (Gao et al., 2023). The function of EPS in promoting biofilm formation is mainly through the following aspects: adhesion, intercellular aggregation, biofilm cohesion, barrier protection, and nutrient support (Simon-Soro and Mira, 2015). This complex three-dimensional biofilm structure creates a unique microenvironment that shields microbes from environmental stressors, such as host immune responses and pharmaceuticals, while also preventing the dissemination of acids that can result in enamel demineralization and subsequently promote the development of tooth decay (He et al., 2016). Meanwhile, the biofilm structure hinders the penetration of drugs, making it difficult for conventional antibacterial agents to exert their effects (Cugini et al., 2019).

Traditional preventive and therapeutic strategies for dental caries primarily involve mechanical interventions, antibiotics, natural plant extract therapy, and the administration of fluoride (Alshahrani and Gregory, 2020; Sun et al., 2021). However, each of these methods has its limitations (Guo et al., 2015). Mechanical therapy is limited by human will and the effect is superficial. Numerous antibiotics may disrupt the natural balance of the bacterial community, which can lead to enhanced resistance among pathogenic bacteria. Moreover, excessive fluorine usage may result in chronic fluorosis. Consequently, seeking more efficient, quick, and safe approaches to inhibit bacterial biofilm formation is essential.

Probiotics, defined as live microorganisms, are known to offer health advantages to hosts when consumed in appropriate quantities (Yeun and Lee, 2015). Currently, *Lactobacillus* is the most studied and applied probiotics. Long-term studies found that *Lactobacillus* could prevent caries by producing metabolites including lactic acid, peroxide, bacteriocin, and proteinaceous compounds, impeding adhesion and colonization, and being effective in downregulating the expressions of virulence genes linked to biofilm formation (Wasfi et al., 2018; Zhao et al., 2023; Zhang et al., 2024).

The cell-free supernatant (CFS) of microbial culture medium is the metabolites produced during microbial growth and residual nutrients in the culture medium. Studies have shown that substances that exert an antibacterial effect in the CFS include lactic acid, acetic acid, hydrogen peroxide, long-chain fatty acids and their esters, and protein compounds (van Zyl et al., 2020; Mani-Lopez et al., 2022). It was reported that the CFS of *Lactobacillus rhamnosus* contained small cyclic peptides that could inhibit the biofilm formation of *S. mutans* (Niranjan et al., 2024).

*Lactobacillus salivarius* is commonly found in human saliva, characterized by its ability to generate organic acids through carbohydrate fermentation, which inhibits the proliferation of surrounding microbes (Kõll-Klais et al., 2005). Because of this antagonistic property, numerous studies have revealed that they could be used to treat periodontal disease and peri-implant diseases, control body weight, and improve the host immunity (Mulla et al., 2021; Chen et al., 2022).

The limited number of studies about the specific mechanism of the probiotic *L. salivarius* against *S. mutans* in cariogenic biofilms prompted us to address this problem. Therefore, this article aimed to evaluate the influence of the *L. salivarius* ATCC11741 supernatant on cariogenic biofilms, explore the potential mechanisms through the supernatant that may intervene in cariogenic biofilms, analyze the functional substances of the supernatant, and evaluate the effect of anti-caries in animal models.

## 2 Materials and methods

### 2.1 Bacterial strains and culture conditions

Strains of *S. mutans* (ATCC 25175) and *L. salivarius* (ATCC 11741) were sourced from the China General Microbiological Culture Collection Center (CGMCC; Beijing, China). *L. salivarius* was grown in de Man–Rogosa–Sharpe (MRS) broth and *S. mutans* was cultured with brain–heart infusion (BHI) broth at 37°C under aerobic and microaerophilic conditions, with all strains stored in the broth containing 30% glycerol at −80°C routinely and were subsequently incubated on appropriate agar plates for 24 h, followed by 2% (v/v) inoculation in the relevant broth at 37°C for an additional 18 h before experimental use.

### 2.2 Preparation of cell-free supernatant

The CFS of *L. salivarius* was manufactured following a modified protocol established by Liang (Liang et al., 2023). In summary, *L. salivarius* was regulated to a concentration of 1×10^7^ CFU/mL during the late logarithmic growth phase and subsequently incubated for 24 h at 37°C. After the bacterial culture, the spent culture underwent centrifugation (5,000×*g*, 10 min, 4°C), and the obtained supernatant was further filtered with a 0.22-µm filter to acquire the CFS.

### 2.3 Biofilm formation assay

An overnight culture of *S. mutans* was diluted to a predetermined final concentration of 1.0×10^6^ CFU/mL in BHI broth enriched with 1% sucrose. This diluted culture was then dispensed into a 96-well microtiter plate at a volume of 200 μL, either with or without the increase of CFS, and incubated for 24 h. The final CFS concentrations, both treated and untreated, ranged from 12.5% to 100% (v/v). For different time treatment, a certain amount of CFS was added into the 96-well microtiter plate at 0, 6, and 12 h, respectively, at 37°C for 24 h. The method of mediating the biofilm by CFS at 24 h was as follows: 200 μL of suspension of *S. mutans* was added to each well of a 96-well plate and then cultured for 24 h, the biofilm was washed with phosphate-buffered saline (PBS) two times, and then 100 μL of CFS was added and the culture was continued for another 24 h. A crystal violet staining assay was conducted to evaluate how effectively the CFS inhibited biofilm formation by *S. mutans* (Wasfi et al., 2018). Specifically, after removing the culture supernatant, the biofilm was washed with PBS and fixed in methanol for 30 min. Afterward, the wells were stained with 0.1% crystal violet solution for 30 min, and then dissolved in 33% glacial acetic acid over 30 min until fully solubilized. Finally, optical densities were recorded at 575 nm using a microplate reader (the amount of biofilm formation). As a negative control, CFS was replaced by MRS broth.

### 2.4 Biofilm microstructure observed using scanning electron microscopy

An overnight culture of *S. mutans* was maintained in BHI broth and subsequently diluted to 1.0×10^7^ CFU/mL (with 1% sucrose). A sterile cover slide was placed into the wells of a 24-well plate. In each well, 800 μL of the *S. mutans* suspension was combined with 160 μL of CFS or MRS broth and incubated under anaerobic conditions for 24 h. The cover slides were carefully rinsed three times using PBS, fixed, and prepared for scanning electron microscopy (SEM) observation (Hitachi, SU8100) following an established protocol (Liu et al., 2021).

### 2.5 Fluorescence staining for observing the proportion of live and dead bacteria in biofilm

Suspensions (1.75 mL) and 350 μL of CFS were added into the confocal dishes and cultured at 37°C for 24 h. The biofilm was subjected to staining for 30 min and subsequently observed using a confocal laser scanning microscope (CLSM) according to the BBcell Probe™ live/dead bacterial staining kit. In the control conditions, MRS broth served as a substitute for the CFS.

### 2.6 Physical and chemical properties of active components in CFS

#### 2.6.1 Temperature stability

The CFS was treated at 50°C, 60°C, 70°C, 80°C, 90°C, and 100°C for 30 min in a dry thermostatic metal bath, respectively. The activity of inhibiting biofilm formation mediated by these CFSs was compared.

#### 2.6.2 pH and enzymatic stability

The pH of the supernatant was adjusted to 6.5 using 1 mol/L NaOH, maintained for 1 h, and then readjusted to the initial pH value (3.9) with 1 mol/L HCl. In addition, 5 mg/mL of catalase and 1 mg/mL of proteinase K (Solarbio, Beijing, China) were added to the CFS for enzymatic stability. Untreated CFS was used as control.

#### 2.6.3 Non-targeted metabolome analysis

CFS and MRS culture medium were respectively divided into six biological replicates for LC-MS analysis conducted by Majorbio (Majorbio Biotech Co., Ltd., Shanghai, China) for non-targeted metabolomic evaluation, in accordance with the method depicted in the literature with minor modifications (Hou et al., 2021). A volume of 200 μL of the sample underwent ultrasonication and was obtained using 800 μL of mixture of methanol and acetonitrile (1:1, v/v) that contained an internal standard and subjected to ultrasonication at 40 kHz (5°C, 30 min). The samples were frozen at −20°C for half an hour, centrifuged for 15 min, and then evaporated with a stream of N2 gas. The resultant samples were reconstituted in 120 μL of an acetonitrile:water solution (1:1, v/v) and were ultrasonicated again at 40 kHz (5°C, 10 min). After being centrifugated at 13,000×*g* (4°C, 10 min), we transferred the obtained supernatants into sample bottles in preparation for subsequent LC-MS/MS analysis. Furthermore, to maintain analytic stability, quality control (QC) samples were created by merging 20 μL of specimen from every sample.

The analysis was conducted via LC-MS/MS utilizing the UHPLC-Q Exactive HF-X system (Thermo Fisher, Waltham, MA, USA). Progenesis QI v3.0 was employed for processing the raw data (Waters Corporation, Milford, USA). To enhance the distinctions among the groups and identify variables of class separation, a supervised clustering method known as partial least squares discriminant analysis (PLS-DA) was implemented. We calculated variable importance in the projection (VIP) values to demonstrate the roles of different variables within the PLS-DA model. The metabolites were annotated according to KEGG for the analysis of metabolic pathways as well as for the classification of compounds. The differential metabolites were categorized using the HMDB database. A difference in metabolite production between the two groups was considered significant if *p* < 0.05 and VIP > 1.

### 2.7 Transcriptome analysis by RNA-seq

*S. mutans* were cultured statically in six-well polystyrene plates for 24 h, allowing loosely attached bacterial cells to be gently rinsed off and then the biofilms were scraped off and approximately 50 mg of biofilm mass was collected by centrifugation and frozen at −80°C until used. Library construction, sequencing, and analysis services were provided by GENEWIZ Life Sciences (Suzhou, China). Each group for transcriptomics had three replicates. The data presented in the study are deposited in the NCBI repository, accession number PRJNA1219341.

### 2.8 Total bacterial RNA extraction and quantitative real-time polymerase chain reaction

The biofilm formation assay adopted the same preparation as before. Following incubation, the culture suspension from the wells was discarded. The plate wells underwent two washes with sterile saline, after which biofilm was scraped and suspended in saline for transfer to a centrifuge tube. The RNAprep pure Cell/Bacteria Kit (Tiangen Biotech; Beijing; China) was used for total RNA extraction according to the instructions of this kit. RNA concentration and purity were assessed using the ND-1000 spectrophotometer. Primer [Sangon Biotech Company (Shanghai, China)] sequences are listed in **Table 1**. The RNA was reversely transcribed into cDNA with the NovoScript Plus All-in-one 1st Strand cDNA Synthesis SuperMix (gDNA Purge) kit (Novoprotein, E047, Suzhou, China), subsequently utilizing the NovoStart SYBR qPCR SuperMix Plus reagent (Novoprotein, E096, Suzhou, China). The internal reference was 16S rRNA. The gene transcription level was evaluated using the 2^−ΔΔCT^ methodology.

**Table 1:** The table of primer sequences.

Primers	Sequences (5′–3′)
16S rRNA	F: CCTACGGGAGGCAGCAGTAG R: CAACAGAGCTTTACGATCCGAAA
LrgB	F: GGCAAAAGGATTGGGAACTGATG R: TGGAACGGCAAAGGCAATGG
DexA	F: AGGGCTGACTGCTTCTGGAGT R: AGTGCCAAGACTGACGCTTTG
Ldh	F: TCCTGTTGGAGGTGGCATTC R: TGCTGTACCCGCATTCCATT

### 2.9 Inhibitory effect of *L. salivarius* on *S. mutans* virulence *in vivo*

#### 2.9.1 Animals and general procedures

**Figure 1:**
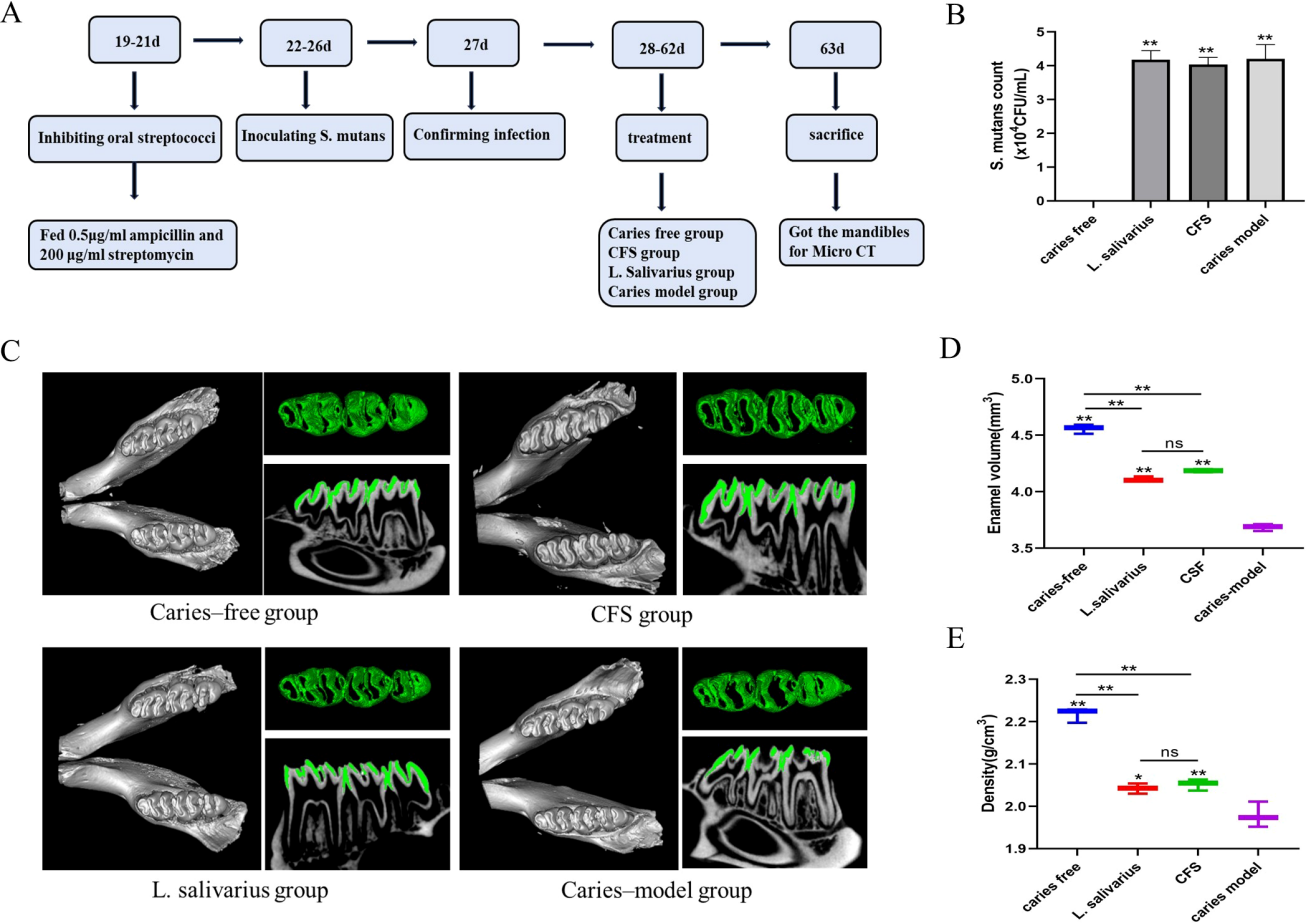
Flowchart of animal model **(A)**. *S. mutans* count from different treatment groups **(B)** ,**p < 0.01 significantly different from the caries free group. Data were expressed as mean ± standard error of the mean (*n* = 3). 3D micro-CT image of mandibular molars, separated enamel (green), and corresponding 2D scale sagittal slice of the same molar (enamel is green) in each group **(C)**. Volume of enamel of mandibular molars **(D)** **p < 0.01 significantly different from the caries-model group; ns, not significant. Density of enamel of mandibular molars **(E)**, *p < 0.05, **p < 0.01 significantly different from the caries-model group; ns, not significant.

The design of animal experiments was similar to the description by previous literature (Zhang et al., 2020). The flowchart of the animal model is shown in **Figure 1A**. Male SPF Sprague–Dawley rats (18 days old) were bought from the Jinzhou Medical University experiment animal center. The experiment received approval from the school’s Animal Protection and Institutional Committee (approval number: 230078) in accordance with the national animal protection guidelines. All laboratory animals were operated on under anesthesia and all efforts were made to reduce pain, suffering, and mortality. A total of four groups of rats (*n* = 3) were randomly assigned, including two treatment groups (*L. salivarius* suspension and CFS), as well as the caries-free and caries model groups. The rats in the caries-free group were given normal diet and distilled water during the whole experiment. Other groups were offered a cariogenic diet 2000 (obtained from Jiangsu Xietong Pharmaceutical Bio-engineering Co., Ltd.) and water containing 5% sucrose. To suppress the oral bacteria group, 0.5 μg/mL of ampicillin and 200 μg/mL of streptomycin were administered for 3 days prior to modeling. Over a period of 5 days starting at the experiment’s commencement, three groups of rats used for caries modeling were infected with *S. mutans*. This was achieved by saturating sterile cotton swabs with 1 mL of an *S. mutans* culture (10^8^ CFU/mL) and applying the suspension into each quadrant of the rat’s mouth for 15 s. After the application of the tooth coating, dietary and water access was restricted for 2 h to facilitate the colonization of the microorganisms. At the age of 27, 100 μL of saliva was collected and coated on a plate for culture for detecting the colonization of *S. mutans* and recording the colony-forming units (CFU) for verifying the establishment of tested strains. The treatment groups were then administered 1 mL of *L. salivarius* suspension or CFS one time per day until the experiment’s conclusion (from days 28 to 63). The rats’ weights were recorded weekly, and the weight gain was calculated.

#### 2.9.2 Micro-CT analysis

After successful modeling, SD rats were euthanized. All the mandibles were harvested, fixed with 4% paraformaldehyde, and then imaged with a VENUS Micro CT instrument (Kunshan, China). 3D pictures were created utilizing AVATAR 1.5.0 software. The enamel was separated from the mandible with fixed thresholds and the mineral density and volume of the enamel were evaluated after correction for hydroxyapatite criteria.

### 2.10 Statistical analysis

Each experiment was performed three times, and all results were expressed as mean ± standard deviation. One-way analysis of variance (ANOVA) was used for statistical analysis along with Dunnett’s test through GraphPad Prism version 8.0.1. Subsequently, all pairs of mean comparisons were assessed using the *post-hoc* Tukey method. *p* < 0.05 was considered statistically significant, while *p* < 0.01 was considered highly statistically significant.

## 3 Results

### 3.1 Antibiofilm effect of CFS of *L. salivarius* against *S. mutans*

**Figure 2:**
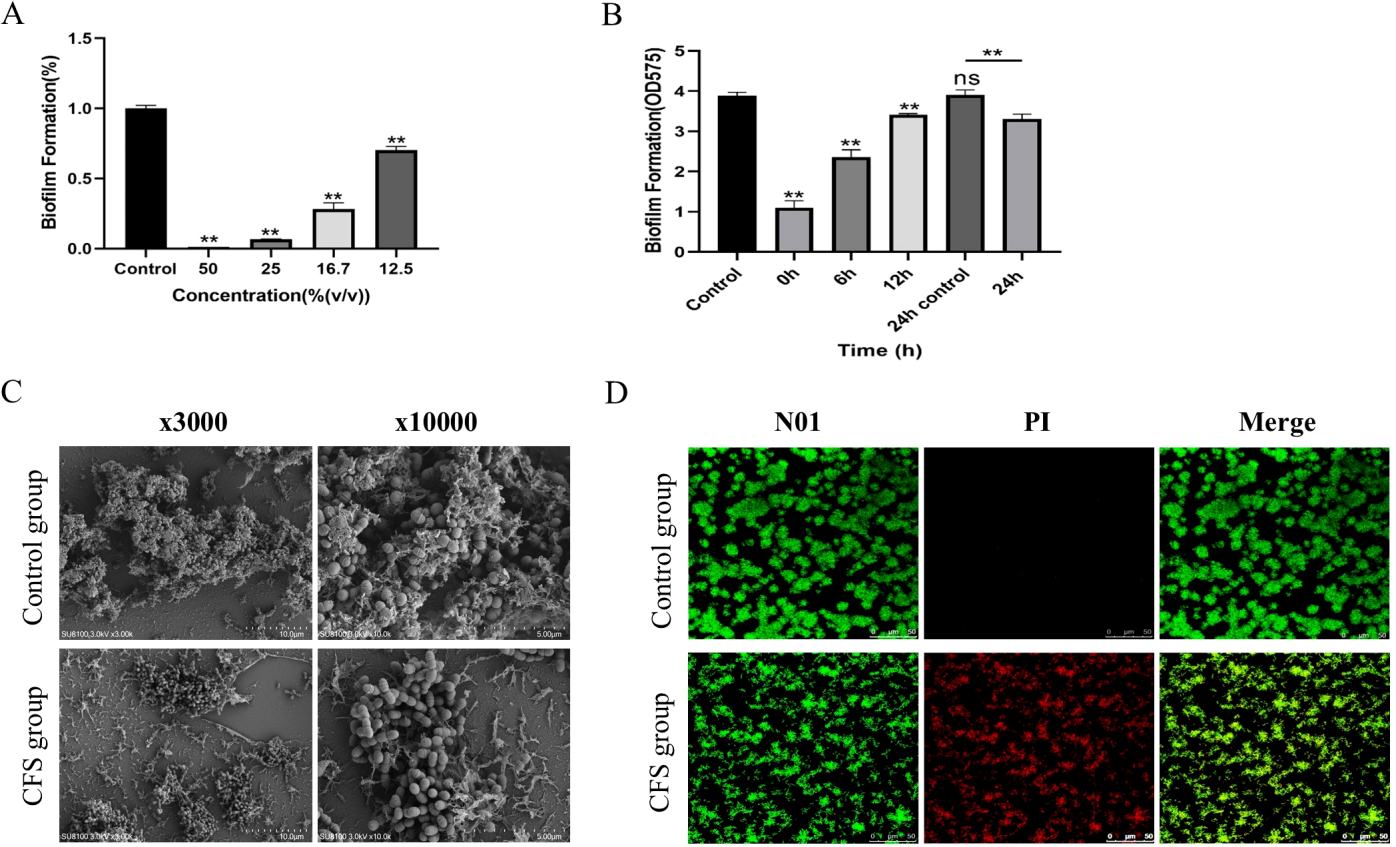
Antibiofilm effect of CFS against *S. mutans* **(A)**. Biomass of the biofilm mediated by CFS at different times **(B)**. The data are presented as the means ± SD. **p < 0.01, compared with the control group; ns, not significant. Effect of CSF on the structure of *Streptococcus mutans* biofilm **(C)**. Effect of CSF on the activity of *Streptococcus mutans* biofilm **(D)**. Red, non-viable cells; green, viable cells; yellow, overlap of non-viable and viable cells. Bar = 50 μm.

As illustrated in **Figure 2A**, all CFSs at varying concentrations demonstrated efficacy in suppressing the biofilm formation of *S. mutans*. Specifically, CFS could inhibit 99% of *S. mutans* biofilm formation when used at concentrations of 50% (v/v). When the CFS was diluted to the concentration of 25%, the effect of biofilm formation decreased to 93.4% (*p* < 0.05). As the CFS was diluted to 12.5%, the inhibition decreased to 28.6% (*p* < 0.01).

The biofilm formation process included four essential time points: 0 h, where bacteria initially adhered; 6 h, marking the initial colonization of bacteria; 12 h, indicating early biofilm development; and 24 h, reflecting mature biofilm formation. As **Figure 2B** shows, the amount of biofilm mediated by each time point decreased significantly (*p* < 0. 01); the 24-h-mediated biofilm showed a certain reduction in biofilm volume compared with the 24-h control biofilm (*p* < 0.01). The results showed that the CFS of *L. salivarius* had a strong inhibitory effect in the early stage of biofilm formation, but had a slightly destructive effect on middle and mature stage of biofilm formation.

At a dilution of 16.7% for the CFS, the SEM micrograph presented in **Figure 2C** demonstrated that a compact biofilm typical for *S. mutans* displayed a network-like composition identified as EPS. However, the biofilm architecture of *S. mutans* added with CFS appeared significantly more dispersed, exhibiting fewer micro-colonies on the surface compared to that of *S. mutans* alone, with a decrease in the quantity of EPS. Biofilms were created in the presence of CFS following 24 h of cultivation and analyzed by a confocal laser scanning microscope (**Figure 2D**). The images demonstrate fluorescence intensities in green (live bacteria) and red (dead bacteria). The control group revealed that the biofilm was distributed uniformly, accompanied by a comparatively dense structure and total surface coverage. As shown in the CFS treatment group, the biofilms seemed significantly more dispersed and notably looser. The surface area that the biofilm occupied decreased because of CFS, leading to a marked reduction of biofilm biomass.

### 3.2 Analysis of antibiofilm components in CFS

**Figure 3:**
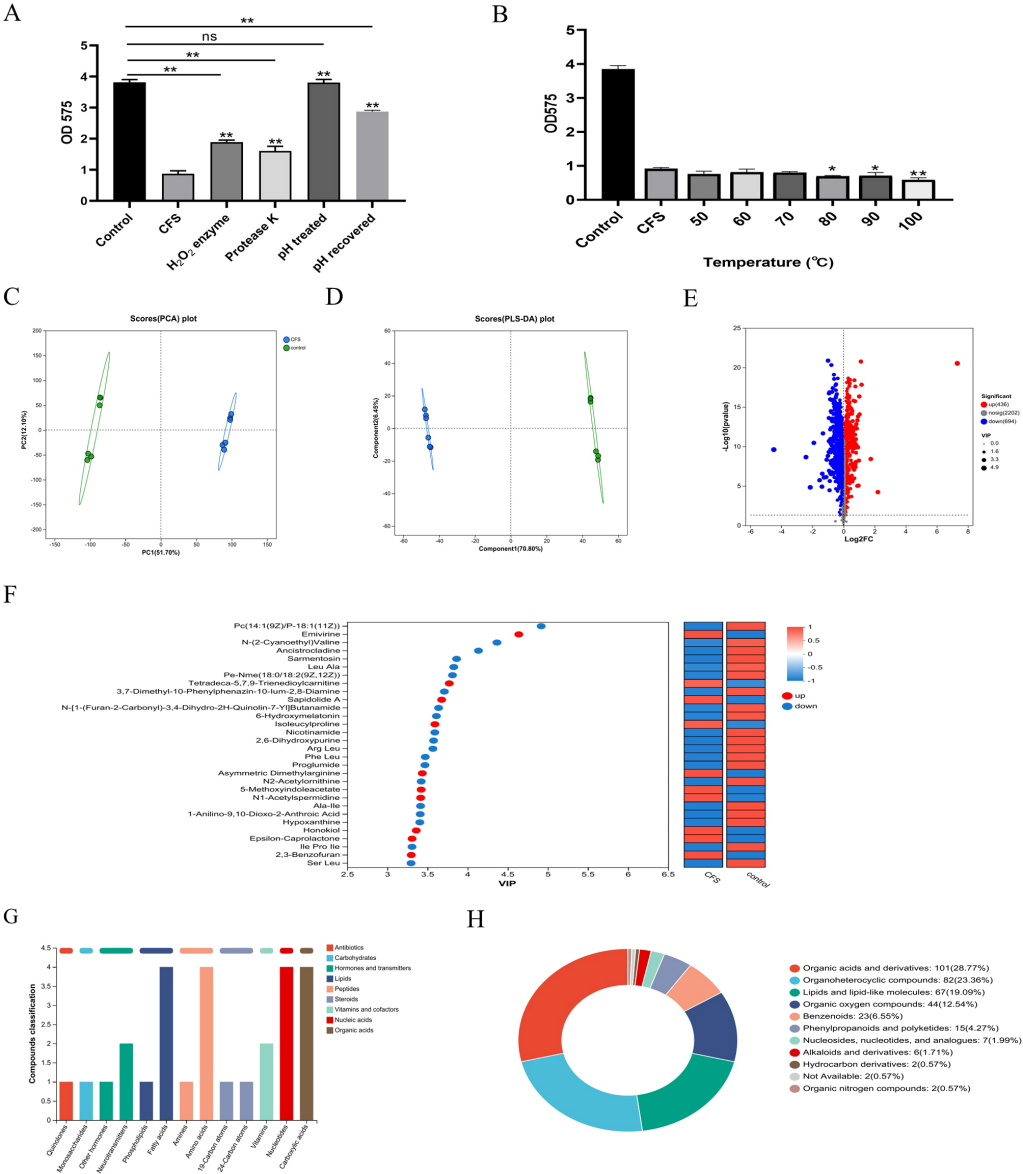
The inhibition effect on the biofilm of the CFS after different treatments. pH and enzyme **(A)** and temperature **(B)**. *p < 0.05, **p < 0.01 significantly different from the control group; ns, not significant. The metabolites in the CFS group compared to those in the control group. Principal component analysis (PCA) score plot of metabolite profiles from the treated and control groups **(C)**. Partial least squares discriminant analysis (PLS-DA) score plot of metabolite **(D)**. Volcano plot of the metabolites from CFS and control **(E)**. Variable importance for the projection (VIP) score calculated by PLS-DA **(F)**. KEGG compound analyses of upregulated metabolites **(G)**. HMDB compound classification diagram **(H)**.

Literature indicated that the antimicrobial components found in the CFS from *Lactobacillus* consist of organic acids, hydrogen peroxide, and bacteriocin (Vahedi Shahandashti et al., 2016; Liu et al., 2020). Bacteriocin is a protein synthesized by ribosomes, a metabolite secreted by bacteria during their reproductive process, possessing antibacterial properties (Kumariya et al., 2019). To preliminarily investigate the characteristics of the primary antibacterial agent, three portions of CFS were exposed to catalase and proteinase K, and pH was adjusted to neutral levels. After being treated with catalase and proteinase K, *S. mutans* showed a reduced but not lost antibiofilm effect. These findings suggested that hydrogen peroxide and a protein-like substance could serve as the principal antibiofilm agent in the CFS of *L. salivarius*. Additionally, this research revealed that the pH of the obtained CFS measured at 3.9. After adjusting the pH to 6.5, the CFS lost its antibiofilm capability; meanwhile, when the pH returned to the original value, the antibiofilm effect of CFS had not been fully restored (**Figure 3A**). As a result, we conducted further examinations to identify the main antibiofilm constituents of CFS. After the CFS was treated at different temperatures, there were no notable changes in its ability to inhibit biofilm formation. Even after 30-min treatment at 100°C, it still had the effect of inhibiting biofilm, and interestingly, it was slightly increased compared to that without treatment after 80°C (**Figure 3B**). This indicates that the active components of the CFS were thermally stable.

To analyze the effective components of CFS that enforce antibacterial properties, the extracellular metabolite profiles were compared between MRS and CFS through metabolomic analysis following a 24-h incubation period. The principal component analysis (PCA) and PLS-DA models highlighted significant metabolite differences between the two sample groups, all within the 95% confidence interval (**Figures 3C, D**). In the presence of the CFS, 436 metabolites were upregulated, while 694 were downregulated (*p* < 0.05, VIP > 1) compared with those in the presence of MRS broth (**Figure 3E**). There was a significant difference between the fermentation broths before and after cultivation, indicating that effective substances that may inhibit biofilm formation have been produced during the fermentation process. The VIP plots (**Figure 3F**) indicated that certain identified metabolites contributed to class differentiation. KEGG compound analysis of the upregulated metabolites was conducted, mainly including fatty acids, amino acids, nucleotides, and carboxylic acids (**Figure 3G**). The upregulated metabolites predominantly included various compounds including organic acids and derivatives, lipids and lipid-like substances, organoheterocyclic entities, organic oxygen compounds, and benzenoids were produced, as indicated by the HMDB compound classification analysis (**Figure 3H**). This section explored the characteristics of the effective components in CFS, which were thermally stable and possible protein and hydrogen peroxide-like substances and have a relatively narrow pH tolerance range, possibly being a type of organic acid or a substance that functions under acidic conditions. Furthermore, by combining with the non-targeted metabolomics method based on LC-MS to identify the differential metabolites between the CFS and the initial culture medium, we found that they were mainly fatty acids, amino acids, nucleotides, and carboxylic acids.

### 3.3 Transcriptome analysis by RNA-seq and analysis of qRT-PCR results

Given that CFS led to notable inhibition of biofilm formation by *S. mutans*, we conducted transcriptome analysis on both *S. mutans* treated with CFS and those under control conditions to monitor genome-wide gene expression alterations caused by CFS.

**Figure 4:**
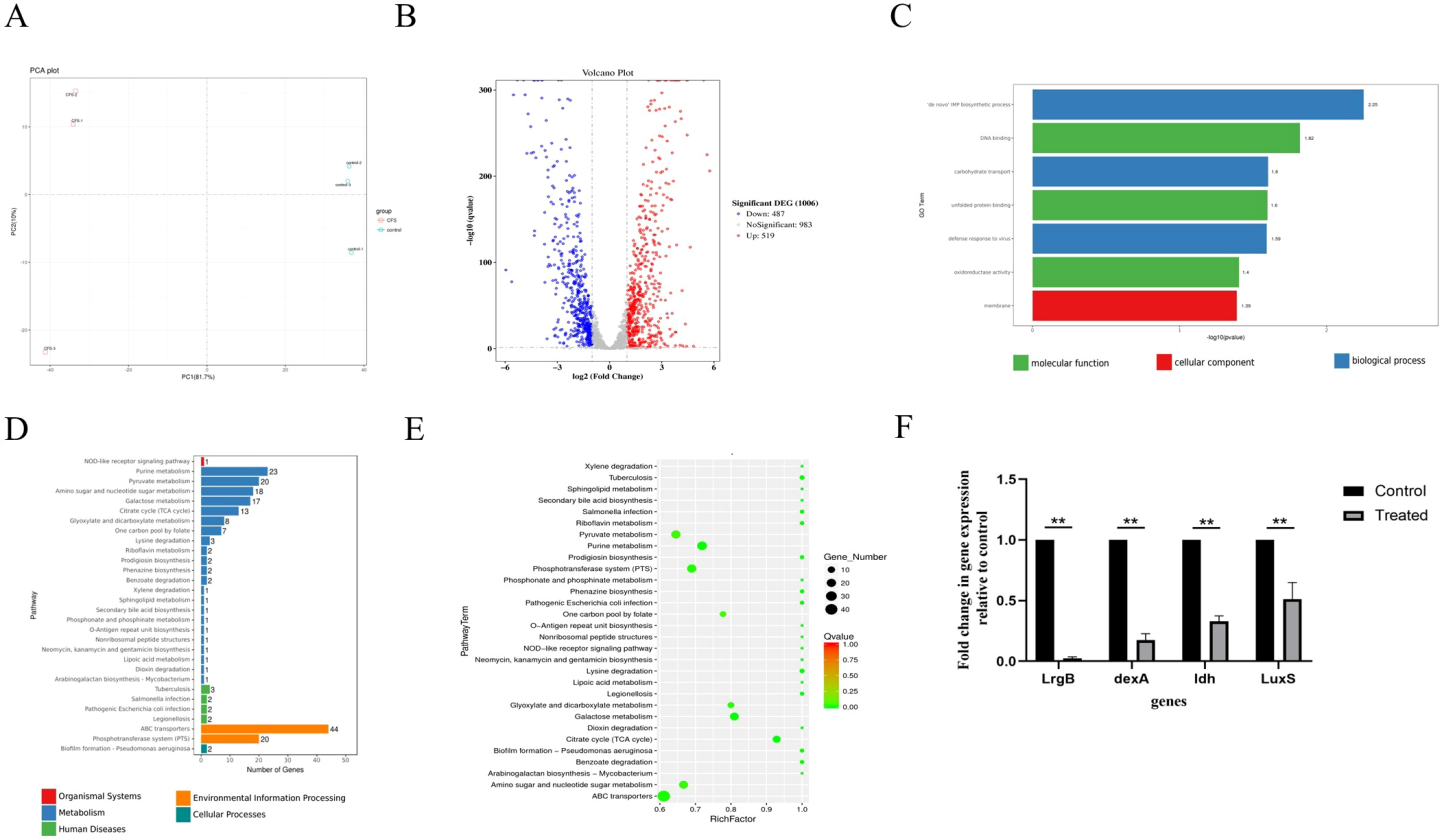
Effects of CFS through RNA-Seq analysis in *S. mutans* biofilms. Principal component analysis (PCA) score plot from the treated and control groups **(A)**. Volcano plot illustrating genes with significant expression differences of *S. mutans* biofilms in the CFS group **(B)**. GO annotation analysis of differentially expressed genes **(C)**. KEGG pathway analysis of differentially expressed genes **(D)**. KEGG rich analysis of differentially expressed genes **(E)**. Validation of differentially expressed genes using qRT-PCR **(F)** **p < 0.01 significantly different from the control group. Fold change = 2^−ΔΔCT^. Fold change >1 indicates upregulation, <1 indicates downregulation.

PCA showed that all biological replicates clustered closely, suggesting that gene expression underwent significant alteration due to CFS treatment compared to untreated controls, accounting for the 81.7% variance observed in the overall dataset (**Figure 4A**). The differentially expressed genes (DEGs) were identified using a modified *p*-value threshold of < 0.01 observed through the control and CFS groups. As depicted in the volcano plot and heatmap in **Figure 4B**, 487 genes were found to be upregulated, while 519 exhibited downregulation. DEGs were annotated, functionally utilizing KEGG enrichment analysis. The GO enrichment assessment concerning molecular functions, cellular components, and biological processes revealed the involvement of these DEGs in DNA binding, oxidoreductase activity, membrane, *de novo* IMP biosynthetic process, and carbohydrate transport (**Figure 4C**). The functional annotation of DEGs was carried out along with KEGG pathway and enrichment analysis (**Figures 4D, E**). Furthermore, 30 significant KEGG pathways were revealed, demonstrating potential links to the metabolic processes of *S. mutans*, including pathways related to pyruvate metabolism, purine metabolism, amino sugar and nucleotide sugar metabolism, galactose metabolism, and the TCA cycle. Certain pathways, especially pyruvate metabolism, the phosphotransferase system, and ATP-binding cassette (ABC) transporters, play vital roles in biofilm formation.

*S. mutans* possesses 14 phosphoenolpyruvate-dependent phosphotransferase systems (PTSs) and two ABC transporters, indicating that it can transport various sugars to meet its metabolic needs and adapt to complex and changing environments. Transcriptome analysis indicated a significant reduction in the key gene expression associated with carbohydrate metabolism because of CFS (**Tables 2**, **3**). Selected genes related to two-component signal transduction systems (*ciaH, ciaR, lytS*, and *lytR*), polyketides/non-ribosomal peptides (*mub gene clust*), acid stress response (*aguA* and *TreR*), quorum sensing gene (*LuxS*), and exopolysaccharide-formation gene (*dexA*) were significantly downregulated and are presented in **Table 4**, which could inhibit oxidative stress and attenuate the virulence of *S. mutans*.

**Table 2:** Differentially expressed genes in *S. mutans* upon CFS treatment related to PTSs.

Gene ID	Gene name	log2FoldChange	Gene product description
gene-D820_RS08610	D820_RS08610	−4.35039416	Metal ABC transporter ATP-binding protein
gene-D820_RS08600	D820_RS08600	−5.299646868	Metal ABC transporter substrate-binding protein
gene-D820_RS08605	D820_RS08605	−4.680503916	Metal ABC transporter permease
gene-D820_RS02670	D820_RS02670	−3.320788871	Extracellular solute-binding protein
gene-D820_RS04680	D820_RS04680	−2.095828916	ABC transporter permease/substrate-binding protein
gene-D820_RS02655	ugpC	−2.274872059	sn-glycerol-3-phosphate ABC transporter ATP-binding protein
gene-D820_RS01050	D820_RS01050	−1.381214985	MetQ/NlpA family ABC transporter substrate-binding protein
gene-D820_RS02665	D820_RS02665	−2.14457111	Sugar ABC transporter permease
gene-D820_RS04675	D820_RS04675	−1.977690467	ABC transporter ATP-binding protein
gene-D820_RS05590	msmE	−2.511339465	Sugar-binding protein MsmE
gene-D820_RS04570	D820_RS04570	−1.468165245	BMP family protein
gene-D820_RS08355	D820_RS08355	−1.831419345	Peptide ABC transporter substrate-binding protein
Gene-D820_RS0109815	D820_RS0109815	−1.45009996	Energy-coupling factor transporter ATPase
gene-D820_RS08420	D820_RS08420	−1.592411347	Amino acid ABC transporter ATP-binding protein
gene-D820_RS02660	D820_RS02660	−2.178411965	Sugar ABC transporter permease
gene-D820_RS05585	D820_RS05585	−1.94908875	Sugar ABC transporter permease
gene-D820_RS08415	D820_RS08415	−1.353656759	ABC transporter substrate-binding protein/permease
gene-D820_RS05580	D820_RS05580	−1.365270344	Carbohydrate ABC transporter permease
gene-D820_RS00140	D820_RS00140	−1.273980434	Energy-coupling factor transporter transmembrane protein EcfT
gene-D820_RS07555	D820_RS07555	−1.06599486	ABC transporter permease
gene-D820_RS05570	ugpC	−1.16129183	sn-glycerol-3-phosphate ABC transporter ATP-binding protein UgpC
gene-D820_RS07560	D820_RS07560	−1.092495506	ABC transporter ATP-binding protein
gene-D820_RS06575	D820_RS06575	−1.011033093	ABC transporter ATP-binding protein

**Table 3:** Differentially expressed genes in *S. mutans* upon CFS treatment related to ABC transporters.

Gene ID	Gene name	log2FoldChange	Gene product description
gene-D820_RS00965	D820_RS00965	−4.232719052	PTS fructose transporter subunit IIA
gene-D820_RS01260	D820_RS01260	−3.270871468	PTS sugar transporter subunit IIB
gene-D820_RS01255	D820_RS01255	−2.777113939	PTS mannose/fructose/sorbose transporter subunit IIC
gene-D820_RS00970	D820_RS00970	−3.736814618	PTS sugar transporter subunit IIB
gene-D820_RS00975	D820_RS00975	−3.46228586	PTS sugar transporter subunit IIC
gene-D820_RS01250	D820_RS01250	−2.372420573	PTS system mannose/fructose/sorbose family transporter subunit IID
gene-D820_RS09065	D820_RS09065	−3.259542925	Fructose-specific PTS transporter subunit EIIC
gene-D820_RS08290	D820_RS08290	−2.02278172	PTS ascorbate transporter subunit IIC
gene-D820_RS09070	pfkB	−3.715535435	1-Phosphofructokinase
gene-D820_RS06470	ptsP	−1.76668403	Phosphoenolpyruvate–protein phosphotransferase
gene-D820_RS09060	D820_RS09060	−2.97617044	Fructose PTS transporter subunit IIA
gene-D820_RS03005	D820_RS03005	−3.082157304	PTS fructose transporter subunit IIB
gene-D820_RS05630	pfkB	−1.199569385	1-Phosphofructokinase
gene-D820_RS03010	D820_RS03010	−1.39127042	PTS transporter subunit IIC
gene-D820_RS03000	D820_RS03000	−2.51286028	PTS sugar transporter subunit IIA
gene-D820_RS02545	celB	−1.24228573	PTS cellobiose transporter subunit IIC
gene-D820_RS02525	D820_RS02525	−1.420784914	PTS cellobiose transporter subunit IIB
gene-D820_RS03015	lacF	−1.020431306	PTS lactose transporter subunit IIA
gene-D820_RS02535	D820_RS02535	−1.757714575	PTS cellobiose transporter subunit IIA
gene-D820_RS07210	D820_RS07210	−1.182133578	PTS sugar transporter subunit IIB

**Table 4:** Differentially expressed genes in *S. mutans* upon CFS treatment related to stress response, quorum sensing gene, and exopolysaccharide formation.

Gene ID	Gene name	log2FoldChange	Gene product description
gene-D820_RS04535	ciaH	−2.388584358	Three-component system sensor histidine kinase
gene-D820_RS04530	ciaR	−1.660277218	Three-component system response regulator
gene-D820_RS06890	lytS	−1.044404874	Two-component system sensor histidine kinase
gene-D820_RS06895	lytR	−1.490154315	Two-component system response regulator
gene-D820_RS06900	lrgA	−4.847658908	Holin-like protein
gene-D820_RS06905	lrgB	−4.310981884	Antiholin-like protein
gene-D820_RS07385	LuxS	−1.164248994	S-ribosylhomocysteine lyase
gene-D820_RS00575	dexA	−1.002077734	Dextranase
gene-D820_RS03620	mubB	−1.879608552	Mutanobactin A non-ribosomal peptide synthetase
gene-D820_RS03625	mubC	−1.996091508	Mutanobactin A non-ribosomal peptide synthetase
gene-D820_RS03630	mubD	−1.837350518	Mutanobactin A non-ribosomal peptide synthetase
gene-D820_RS03555	mubY	−5.956253759	Mutanobactin A system ABC transporter permease subunit
gene-D820_RS03610	mubH	−1.182104006	Mutanobactin A polyketide synthase
gene-D820_RS03580	mubR	−1.400053226	Mutanobactin A biosynthesis transcriptional regulator
gene-D820_RS08310	aguA	−1.50902996	Agmatine deiminase
gene-D820_RS00580	treR	−1.242887742	Trehalose operon repressor

To confirm the RNA-Seq findings, randomly selected genes underwent quantification through quantitative real-time polymerase chain reaction (qRT-PCR) to assess their transcription levels. As indicated in **Figure 4F**, the genes *lrgB, LuxS, dexA*, and *ldh* showed significant downregulation following treatment with the supernatant, which conformed with the results derived from RNA-seq analysis.

### 3.4 Inhibitory effect of *L. salivarius* on *S. mutans* virulence *in vivo*

Throughout the entire experimental duration, the rats maintained stable health conditions. Weight gain among all groups showed no statistically significant differences (**Table 5**). As shown in **Figure 1B**, in the caries-free group, *S. mutans* was not detected, while the levels of *S. mutans* in the caries model, *L. salivarius*, and CFS groups were approximately 4.0 × 10^4^ CFU/mL after infection for 5 days, demonstrating successful colonization of *S. mutans* within the oral cavities.

**Table 5:** Changes in body weight of SD rats during the experiment.

Group	Weight of SD rats at different periods (g)					
	28 d	35 d	42 d	49 d	56 d	63 d
Caries-free	79.1 ± 1.517	117.1 ± 0.945	139.2 ± 1.417	161.6 ± 0.742	182.1 ± 0.721	223.3 ± 1.627
CFS	77.2 ± 0.907	116.5 ± 0.869	139.9 ± 1.214	60.2 ± 1.229	178.7 ± 1.592	220.9 ± 1.178
*L. salivarius*	80.3 ± 0.624	117.1 ± 0.090	138.2 ± 0.561	160.4 ± 1.186	179.0 ± 1.733	219.7 ± 1.358
Caries-model	79.6 ± 1.444	118.5 ± 1.129	141.3 ± 1.178	158.8 ± 1.503	180.6 ± 1.389	221.1 ± 1.212

In order to improve the visibility of caries site on rat molars, micro-CT was used for 3D reconstructions of the mandibular molars, and the enamel was isolated from the complete mandible with a predetermined threshold. Additionally, the relevant sagittal slice of the homorganic molar was extracted for comparative analysis (**Figure 1C**). From the complete 3D reconstruction of dental hard tissue, the sagittal slice images of the caries model group were compared with those of the caries-free group, in which it was clear that the enamel (green) areas were discontinuous in the presence of caries. Additionally, to quantitatively assess the results from micro-CT, we calculated and analyzed the enamel volume and mineral density of molar teeth across the various experimental groups. The smaller the enamel volume, the more enamel loss and the more severe the caries. As shown in **Figures 1D, E**, the enamel volume and mineral density of the caries model group were obviously less than those in the caries-free group, indicating that the rat caries model was successfully established. The enamel volume and mineral density of the molars treated with CFS were higher than those in the caries-model group and lower than those in the caries-free group (*p* < 0.01). In addition, no significant differences were found in enamel volume and mineral density between the CFS and *L. salivarius* groups.

## 4 Discussion

In the oral cavity, *S. mutans* and *Lactobacillus* are common microorganisms. Similar to the intestinal microbiota, the oral microbiota is also in a dynamic equilibrium. Once this equilibrium is disrupted, cariogenic microorganisms such as *S. mutans* will become dominant, contributing to the formation of a cariogenic biofilm (Hannig and Hannig, 2009). Hence, preventing bacterial biofilm formation is vital for maintaining dental health. In previous investigations (Wasfi et al., 2018; Liang et al., 2023), the inhibition of *S. mutans* biofilms occurred with the addition of the *L. salivarius* supernatant. The pathogenic mechanisms by which the *L. salivarius* supernatant aids in caries prevention and the potential active substances remain unclear. This study demonstrated that the inhibitory effect of the *L. salivarius* supernatant can reduce the biofilm amount and biofilm activity at different time points. It not only significantly reduced the adhesion of initial biofilms, but also has a potential inhibitory effect on 24-h mature biofilms. The biofilm structure after co-cultivation of CFS and *S. mutans* was significantly loose and sparse, which also has a significant destructive effect on the biofilm structure.

*Lactobacillus*, an essential category of probiotics, is widely utilized. The growth and reproduction of harmful bacteria can be inhibited by certain compounds, mainly through their metabolites such as organic acids, bacteriocins, and hydrogen peroxide (Liu et al., 2020). Neutralizing the CFS to pH 6.5 markedly diminished its antimicrobial efficacy, which found that the active components of the CFS could be organic acids or substances that exert an inhibitory effect in acidic environments. The addition of catalase and proteinase K to the CFS resulted in a reduction of its antibacterial activity against *S. mutans*. This indicated that hydrogen peroxide and protein material contribution in antimicrobial activity of the CFS were also important.

Furthermore, the non-targeted LC-MS/MS method was employed to detect the bioactive compounds present in CFS. A mountain of organic acids and derivatives were found from the HMDB analysis. Studies indicated that certain organic acids could impede biofilm formation by certain mechanisms. Among the usual organic acids found in the supernatant of *L. plantarum* CCFM8724, phenolactic acid can significantly suppress the biofilm formation of *S. mutans* and *Candida albicans*. Additionally, phenyllactic acid, a kind of postbiotics, and the secretion of *L. paracasei* ET-22 exhibited significant inhibition of a variety of pathogenic bacteria biofilm formation (Wu et al., 2023). In the characteristic monosaccharides of CFS from *L. salivarius*, sorbitol had been found to reduce acid production and the amount of bacterial biofilm as well as inhibit the acid production of *S. mutans in vitro* (Takahashi-Abbe et al., 2001). Sorbitol has been confirmed to decrease the dual-species biofilm formation of *S. mutans* and *C. albicans*, leading to change in biofilm structure and glucan production (Chan et al., 2020). Therefore, sorbitol was probably an effective substance in CFS of *L. salivarius*. Additionally, we found that the expression of honokiol was upregulated from VIP analysis, which was confirmed to suppress biofilm formation as well as the production of extracellular matrix and lactic acid in *S. mutans* (Ren et al., 2023).

Sugars are the main carbon source for bacteria, which can be used to produce adenosine triphosphate (ATP) and synthesize various cellular components (such as peptidoglycan, fatty acids, and nucleic acids) and intercellular polysaccharide. The primary means of carbohydrate transport in the dental pathogen *S. mutans* occurs through the glycolysis pathway via the PTS system and ABC transporters. *S. mutans* encodes 14 PTSs and two ABC transporters (Kawada-Matsuo et al., 2016; Zeng et al., 2017). Transcriptome analysis of *S. mutans* demonstrated that the expression of major carbohydrate metabolism genes was significantly reduced and influenced by the downregulation of CFS, PTSs for galactitol, cellobiose, fructose, lactose, and mannose. Genes involved in maltose and maltodextrin transport in the ABC transporter system such as *malK, malE*, and *malG* were also downregulated.

Two-component signal transduction system (TCSTS) is a protein phosphorylation signaling pathway widely present in bacteria, which can regulate bacterial gene expression and coordinate various bacterial activities when stimulated by environmental stimuli (Hoch, 2000). TCSTS generally involves a dimerized transmembrane receptor, specifically histidine kinase (HK), along with a cytoplasmic response regulator (RR). The HK protein, situated in the plasma membrane, is capable of sensing specific environmental stimuli, while the RR protein, located in the cytoplasm, responds to these stimuli by modulating gene expression. In *S. mutans*, numerous TCSTS, such as *VicK/VicR*, *CiaH/CiaR*, *LytST*, and *LiaS/LiaR*, have been identified in the genome of *S. mutans* with substantial supporting lines of evidence that are associated with various functions, including acid tolerance, oxidative stress response, and biofilm formation in *S. mutans* (Lévesque et al., 2007; Liu and Burne, 2009). In our study, CFS treatment inhibited the gene expression of *ciaH, ciaR, lytS*, and *lytR* in TCSTS. In summary, our results indicate that the CFS’s effect on reducing the virulence of *S. mutans* is partially influenced by the downregulation of TCSTS involved in signal transduction.

The primary components of the *S. mutans* biofilm include polysaccharides, extracellular DNA (eDNA), and adhesin proteins (Shanmugam et al., 2020). In this study, CFS was found to reduce the mRNA expression levels of *lrgA* and *lrgB*, which have a function in the production of eDNA by regulating cell autolysis and the components of membrane vesicles.

Bacterial quorum sensing (QS) is regularly present in Gram-negative and Gram-positive bacteria, which plays an important role in the information exchange among biofilm bacteria under different stress conditions (Valen and Scheie, 2018). AI-2 molecules, as a messenger molecule of QS, have been identified to play a critical role in the communication processes among *S. mutans*. The protease coded by the gene of *LuxS* is a significant catalyst for the synthesis of AI-2; therefore, the *LuxS* gene serves as a marker for producing this signaling molecule (Schauder et al., 2001). The LuxS/AI-2 QS system is known to play a role in several essential physiological functions in *S. mutans* (Hu et al., 2018). Studies indicated that mutations in *LuxS* hindered biofilm formation, decreasing acid tolerance and acid production (Yoshida et al., 2005). In this article, the gene expression of *luxS* was significantly reduced in the CFS treatment group.

The secondary metabolites of *S. mutans* mainly include bacteriocins and polyketides/non-ribosomal peptides (PKs/NRPs) (Wang et al., 2012; Xie et al., 2017). To date, in *S. mutans*, some of the PKs/NRPs that were identified include mutanobactin, mutanocyclin, and mutanofactin, and these metabolites were relatively synthesized by the *mub, muc*, and *muf* gene clusters. These compounds are involved in various functions, including competition between bacterial species, responses to oxidative stress, biofilm formation, and numerous other physiological activities (Wu et al., 2010; Li et al., 2021). Most mutanobactin operon-related genes were downregulated. Consequently, the reduced expression of these secondary metabolites could significantly impact the biofilm development of *S. mutans*.

## 5 Conclusion

In conclusion, the transcriptomic analysis provided new insights into the mechanism by which the supernatant of *L. salivarius* inhibits *S. mutans* biofilms, including inhibition of phosphoenolpyruvate-dependent phosphotransferase systems, two ATP-binding cassette transporters, two-component systems, PKs/NRPs, acid stress response, QS, and exopolysaccharide formation. In addition, non-targeted LC-MS/MS analysis was employed to discover a variety of potential active compounds present in the CFS of the *L. salivarius* against *S. mutans* biofilm. The above results provide a theoretical basis for further isolation and purification of the *L. salivarius* supernatant and the production and application of active components, as well as the manner and conformation of molecular docking of active components and *S. mutans* targets. Therefore, it has the potential to act as a therapeutic agent for the prevention and treatment of caries.

## Data Availability

The data presented in the study are deposited in the NCBI repository, accession number PRJNA1219341.
